# Efficacy and Safety of Sakurajima Radish in Patients with Metabolic Syndrome: A Phase IIb Randomized, Three-Period Crossover Trial

**DOI:** 10.3390/nu18111801

**Published:** 2026-06-03

**Authors:** Akihiro Tokushige, Yuichi Akasaki, Keisuke Shibata, Takashi Sakoda, Akari Tajima, Takashi Kajiya, Naohiro Shirasawa, Narisato Hamada, Akiko Yoshikawa, Kazuyuki Kubota, Tsuminori Yamashita, Kenjuro Higo, Takuro Kubozono, Kouta Funakoshi, Ryota Kawai, Hisako Yoshida, Ayumi Shintani, Katsuko Kajiya, Mitsuru Ohishi

**Affiliations:** 1 Department of Prevention and Analysis of Cardiovascular Diseases, Graduate School of Medical and Dental Sciences, Kagoshima University, Kagoshima 890-8520, Japan; ohishi@m2.kufm.kagoshima-u.ac.jp; 2 Department of Clinical Pharmacology and Therapeutics, Graduate School of Medicine, University of the Ryukyus, Okinawa 903-0215, Japan; 3 Department of Cardiovascular Medicine and Hypertension, Graduate School of Medical and Dental Sciences, Kagoshima University, Kagoshima 890-8520, Japan; yuichia@m2.kufm.kagoshima-u.ac.jp (Y.A.); k.shibata.magic@gmail.com (K.S.); sakodat225@gmail.com (T.S.); tajimaakari@gmail.com (A.T.); kubozono@m.kufm.kagoshima-u.ac.jp (T.K.); 4 Tenyoukai Central Hospital, Kagoshima 892-0822, Japan; t_kajiya@hotmail.com; 5 Keizankai Kyoritsu Hospital, Kagoshima 890-0069, Japan; shirasawa@kyoritu-keizan.or.jp (N.S.); nariri-hamar@hotmail.co.jp (N.H.); 6 Jinaikai Ueyama Hospital, Kagoshima 890-0073, Japan; akuriko@xg8.so-net.ne.jp; 7 Keishinkai Kubota Internal Medicine Clinic, Kagoshima 892-0871, Japan; kubota-naika@po4.synapse.ne.jp; 8 Tsuminori Internal Medicine Clinic, Kagoshima 890-0105, Japan; pivot@isis.ocn.ne.jp; 9 Jiaikai Imamura General Hospital, Kagoshima 890-0064, Japan; kenjyurouichinaika@yahoo.co.jp; 10 Center for Clinical and Translational Research, Kyushu University Hospital, Fukuoka 812-8582, Japan; funakoshi.kouta.503@m.kyushu-u.ac.jp; 11 Department of Medical Statistics, Graduate School of Medicine, Osaka Metropolitan University, Osaka 545-8585, Japan; ryota.kawai@omu.ac.jp (R.K.); hisako.yoshida@omu.ac.jp (H.Y.); ayumi.shintani@gmail.com (A.S.); 12 Faculty of Agriculture, Kagoshima University, Kagoshima 890-0065, Japan

**Keywords:** metabolic syndrome, flow-mediated dilation, Sakurajima radish, trigonelline

## Abstract

Background/Objectives: We aimed to evaluate the efficacy and safety of a short-term dietary intervention using trigonelline-rich Sakurajima radish on vascular endothelial function in patients with metabolic syndrome (MetS). Methods: In this multicenter, open-label, randomized, three-period crossover phase IIb trial, 21 patients with MetS were assigned to three 14-day sequences (Sakurajima radish powder, Aokubi radish powder, and a usual diet), separated by 14-day washouts. The primary outcome was flow-mediated dilation (FMD). Key Secondary outcomes included blood pressure (BP), urinary nitric oxide metabolites (NOx), and the oxidative stress marker 8-hydroxy-2′-deoxyguanosine (8-OHdG). Results: Sakurajima radish did not improve FMD versus the usual diet (*p* = 0.58) or Aokubi radish (*p* = 0.59), although a significant negative carryover effect following the Aokubi period likely confounded this estimation. Despite successfully stimulating NO production (elevated urinary NOx, *p* = 0.03), the intervention paradoxically increased oxidative stress (elevated 8-OHdG/creatinine, *p* = 0.02) and significantly elevated systolic BP compared with the usual diet (+9.67 mmHg, *p* = 0.03) and Aokubi radish (+8.86 mmHg, *p* = 0.04). Conclusions: Sakurajima radish does not appear to improve endothelial function in patients with MetS within the constraints of this short-term crossover design. Importantly, the unexpected negative carryover effect inherently limits the interpretability of this primary FMD outcome, as it may have masked potential physiological benefits. Despite boosting NO production, the intervention paradoxically exacerbated systemic oxidative stress and elevated systolic BP. These findings suggest that in the pro-oxidant environment of MetS, NO-boosting functional foods may induce unintended adverse hemodynamic responses, underscoring the need for careful risk–benefit evaluation and parallel-group trial designs in this specific population.

## 1. Introduction

Metabolic syndrome (MetS) is a cluster of risk factors, including insulin resistance, abnormal glucose and lipid metabolism, and hypertension, that significantly increase the risk of cardiovascular disease (CVD) [1]. CVD remains the leading global cause of death, with substantial economic and societal burdens owing to prolonged treatment and rehabilitation needs [2]. Among the mechanisms underlying MetS-related vascular dysfunction, endothelial cell impairment, which is characterized by reduced nitric oxide (NO) production, plays a central role in the progression of atherosclerosis [3]. NO bioavailability, a key marker of vascular health, can be measured using flow-mediated dilation (FMD), which is a validated predictor of cardiovascular events [4,5].

Despite the urgent need for interventions targeting vascular dysfunction, current pharmacological options for directly enhancing NO production remain limited. Nutritional approaches, which offer higher adherence and fewer psychological barriers than pharmacological treatments, are promising alternatives [6]. Sakurajima radish (*Raphanus sativus* cv.), characterized by an approximately 60-fold higher trigonelline content compared with Aokubi radish, has shown potential for improving FMD values in a phase I trial involving healthy individuals [7]. In the previous HPLC-based compositional analysis, trigonelline concentrations in Sakurajima and Aokubi radishes were reported as 360 ng/mg and approximately 6.3 ng/mg fresh weight, respectively, corresponding to approximately 2.6 μmol/g and 46 nmol/g fresh weight [7]. While standard-of-care pharmacological interventions (e.g., statins, ARBs) effectively mitigate cardiovascular risk in metabolic syndrome (MetS), they often provide insufficient improvement in established vascular endothelial dysfunction. Trigonelline, a pyridine alkaloid highly concentrated in Sakurajima radish, has emerged as a promising candidate due to its potent, eNOS-dependent NO production mechanism. Previous studies in healthy volunteers have demonstrated that trigonelline successfully translates molecular NO production into macrovascular improvement. However, the vascular microenvironment of MetS—characterized by chronic systemic inflammation and elevated oxidative stress—fundamentally alters the metabolic partitioning of NO. Whether the eNOS-activating potential of trigonelline maintains its therapeutic index in this pro-oxidant milieu, or conversely, triggers maladaptive biochemical pathways, remains a critical unresolved translational question. This study aims to bridge this gap, evaluating whether the systemic NO-boosting effect of Sakurajima radish translates into clinical vascular benefit or paradoxical impairment in patients with MetS. Trigonelline, a pyridine alkaloid, is widely recognized for its diverse pleiotropic properties, exhibiting favorable metabolic and antioxidant roles, such as the attenuation of insulin resistance, lipid-lowering effects, and the scavenging of reactive oxygen species in standard in vitro models. Regarding the mechanism, previous experimental models have revealed that trigonelline potently stimulates NO production via an eNOS-dependent pathway. Specifically, trigonelline increases cytosolic Ca^2+^, leading to the phosphorylation of eNOS at Ser1177 and dephosphorylation at Thr495 [8]. While trigonelline serves as the principal bioactive candidate driving this eNOS-dependent mechanism, the intervention utilizes a whole-food matrix; thus, the synergistic contributions or confounding effects of other radish-derived phytochemicals (e.g., dietary nitrates) to the overall vascular and hemodynamic responses cannot be definitively excluded. While this pathway effectively improves FMD in healthy subjects, its impact under pathological conditions such as MetS remains unknown. Crucially, the dose–response relationship of dietary phytochemicals often exhibits non-linear or hormetic characteristics. While low-to-moderate doses may exert antioxidant and vasodilatory benefits, exposure to high-dose trigonelline may trigger divergent or adverse physiological responses in highly oxidative environments. In patients with MetS, we hypothesize that a robust, high-dose stimulation of NO synthesis could paradoxically overwhelm local antioxidant capacities. Under these conditions, newly synthesized NO may rapidly react with ambient superoxide to generate peroxynitrite, potentially leading to eNOS uncoupling, exacerbated oxidative stress, and adverse vasoconstrictive effects. However, previous findings are limited to a single-arm study in patients with MetS, underscoring the need for randomized controlled trials to establish whether this eNOS-activating potential safely translates into its clinical utility for patients with MetS.

The primary research question of this trial is whether the short-term (14-day) administration of trigonelline-rich Sakurajima radish can improve brachial artery flow-mediated dilation (FMD)—a gold-standard surrogate for vascular endothelial health—in patients with MetS receiving standard-of-care pharmacological therapy. The novelty of this study lies in its shift from demonstrating simple physiological efficacy in healthy subjects to critically interrogating the pharmacological safety and metabolic partitioning of NO in a pathologically altered vascular bed. By employing a three-sequence, three-period crossover design to minimize inter-individual variability, we addressed the clinical uncertainty regarding whether the eNOS-activating potential of trigonelline, previously validated in healthy individuals, can safely translate into therapeutic vascular benefit in patients with MetS. Conversely, we investigated whether the pro-oxidant milieu of MetS triggers a paradoxical, maladaptive hemodynamic response, thereby providing critical evidence to define the risk–benefit profile of NO-boosting functional foods in highly oxidative pathological states.

## 2. Materials and Methods

### 2.1. Study Design

**Figure 1:**
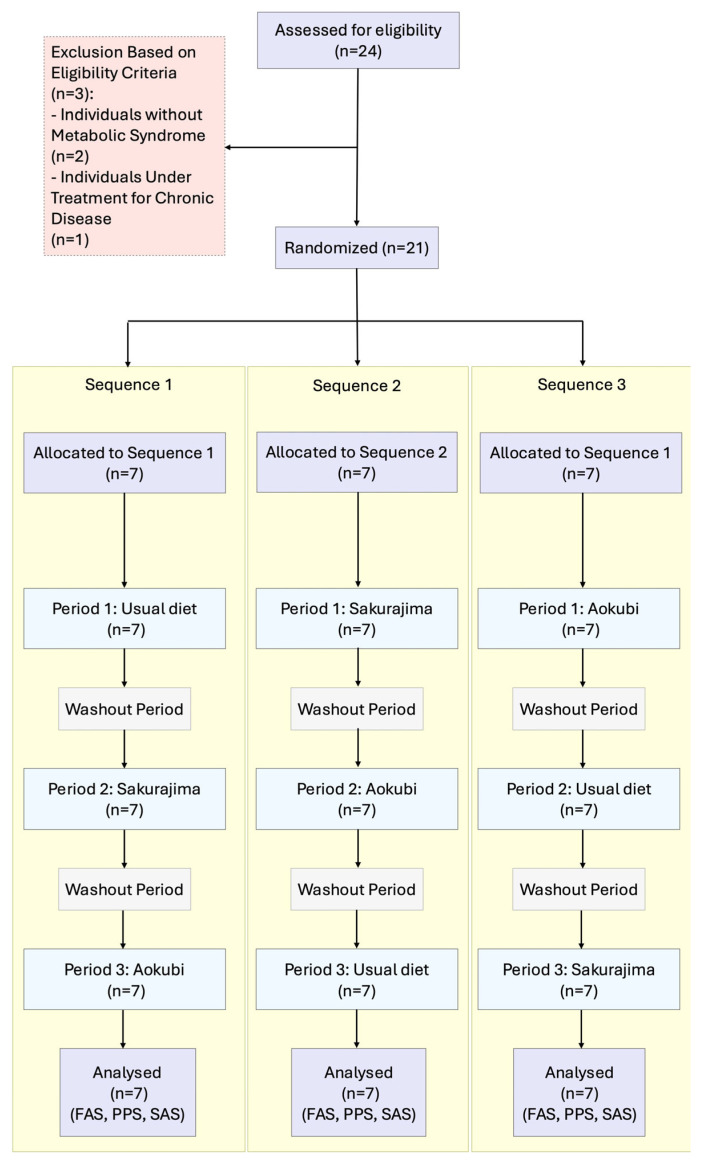
Study participant allocation flowchart.

This study was a multicenter, nonblinded, randomized, three-sequence, three-period crossover phase IIb trial conducted according to the Consolidated Standards of Reporting Trials (CONSORT) guidelines (Figure 1).

The primary objective was the efficacy and safety of Sakurajima radish powder in improving vascular endothelial function in patients with MetS compared with those of Aokubi radish powder and a control period with no dietary intervention.

The crossover design included three 14-day intervention periods (Sakurajima radish, Aokubi radish, and no dietary intervention) separated by a 14-day washout period. This design was specifically chosen to maximize statistical power by neutralizing the substantial inter-individual variability inherent in pathologically diverse MetS cohorts, allowing each participant to serve as their own control. This design minimizes inter-individual variability and controls for potential carryover effects by including sufficiently long washout periods based on trigonelline’s pharmacokinetics.

Furthermore, the inclusion of two distinct control conditions was a critical methodological requirement. The Aokubi radish period served as an active (vehicle) control—accounting for the general “food matrix” effects of consuming a radish (e.g., dietary fiber and baseline nitrates) without the high trigonelline content. Conversely, the usual diet period served as a strict negative control to establish the absolute physiological baseline under free-living conditions. This dual-control structure enables the precise isolation of trigonelline-specific pharmacological effects from general dietary interventions.

Randomization was stratified according to the presence of type 2 diabetes using a computer-generated system with a 1:1:1 allocation ratio.

### 2.2. Participants

This study enrolled adult patients diagnosed with MetS who were recruited from the participating institutions. Detailed eligibility requirements are outlined in the subsequent section.

### 2.3. Eligibility Criteria

#### 2.3.1. Inclusion Criteria

1. Patients aged ≥ 18 years who provided written informed consent.
2. Patients diagnosed with MetS based on established criteria.
3. Patients regularly followed up at the cardiology outpatient clinics of participating institutions.

#### 2.3.2. Exclusion Criteria

1. History of malignant tumors, heart failure, or myocardial infarction.
2. Chronic diseases (e.g., liver or kidney disorders) requiring active treatment.
3. Pregnant, breastfeeding, or planning pregnancy during the study period.
4. Recent smoking or participation in other clinical trials within 3 months prior to enrollment.

#### 2.3.3. Concomitant Medications

Given that the study population comprised patients with MetS, the continuation of baseline pharmacological therapies for underlying conditions—including hypertension, dyslipidemia, and type 2 diabetes (e.g., statins, angiotensin receptor blockers, and oral hypoglycemic agents)—was permitted. However, to prevent these medications from confounding the assessment of vascular endothelial function, participants were strictly required to maintain a stable medication regimen. No initiations of new drugs, discontinuations, or modifications in the dosage of existing cardiovascular or metabolic medications were allowed throughout the entire duration of the crossover trial, including the washout periods.

### 2.4. Intervention

#### Test Foods

**Figure 2:**
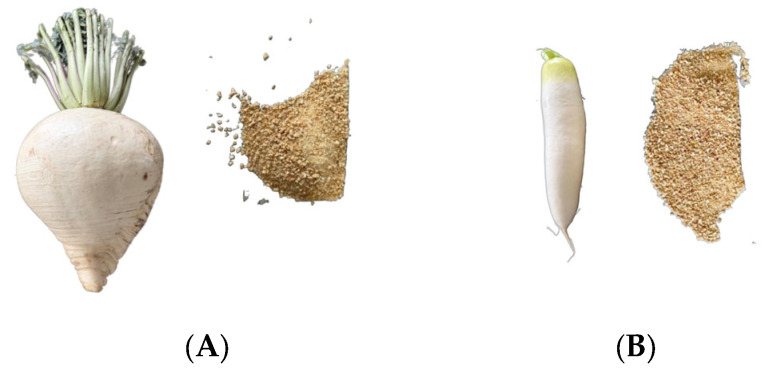
(**A**) Sakurajima radish and powder. (**B**) Aokubi radish and powder.

1. Sakurajima radish powder: Freeze-dried powder prepared from Sakurajima radish (*Raphanus sativus* cv.), which contained approximately 60-fold higher trigonelline content than the other radish varieties (Figure 2A).

2. Aokubi radish powder: Freeze-dried powder prepared from Aokubi radish (*Raphanus sativus*), a widely consumed radish variety in Japan (Figure 2B).

In this study, Sakurajima radish was utilized as a functional food intervention because previous compositional analyses reported that it contains a high concentration of trigonelline compared with Aokubi radish. Specifically, Sasaki et al. reported that raw Sakurajima radish contains 360 ng/mg fresh weight of trigonelline, corresponding to 0.36 mg/g fresh weight or approximately 2.6 μmol/g fresh weight. In contrast, Aokubi radish contains only 1.75% of this amount, corresponding to approximately 6.3 ng/mg fresh weight or 46 nmol/g fresh weight.

In the present trial, participants consumed 10 g/day of freeze-dried radish powder. Based on the reported trigonelline content of fresh Sakurajima radish and the approximately 94–95% water content of fresh radish, the freeze-dried powder was estimated to contain approximately 6.12 mg/g of trigonelline. Thus, the 10 g/day dose was estimated to provide approximately 61.2 mg/day, equivalent to approximately 446 μmol/day, of trigonelline. This value corresponds to the nominal trigonelline intake used in the previous human study of Sakurajima radish. However, because batch-specific quantification of the exact investigational powder lots was not performed in the present trial, this value should be interpreted as an estimated nominal intake rather than a directly measured or confirmed bioavailable dose.

The participants consumed 10 g/day of either radish powder (5 g/packet twice daily) during the intervention period. For both powders, the consumption methods included mixing with water or incorporating them into meals.

### 2.5. Control Period

During the control period, the participants maintained their customary ad libitum diet under free-living conditions with no additional interventions. To minimize dietary confounding, all participants were instructed to maintain their baseline lifestyle, including their general dietary patterns and physical activity levels, throughout the entire study duration (inclusive of washout periods).

### 2.6. Outcomes

#### 2.6.1. Primary Outcome

Improvements in the vascular endothelial function were assessed using flow-mediated dilation (FMD).

#### 2.6.2. Secondary Outcomes

The comprehensive, pre-specified secondary outcomes evaluated in this trial were categorized as follows:

1. Hemodynamic parameters: Systolic and diastolic blood pressure (SBP and DBP) and heart rate.
2. Urinary nitric oxide metabolites and oxidative stress markers: Urinary NOx, urinary 8-OHdG, and serum thiobarbituric acid reactive substances (TBARS).
3. Inflammatory markers: Serum high-sensitivity troponin I (hs-TnI) and pentraxin 3 (PTX3).
4. Vascular function and metabolic parameters: Serum asymmetric dimethylarginine (ADMA) and niacin levels.
5. Pharmacokinetic and tolerability assessments: Serum trigonelline concentrations and sensory evaluation (palatability) of the test foods.

### 2.7. Flow-Mediated Dilation (FMD) Assessment

The FMD measurements followed a standardized protocol. The participants fasted overnight and avoided alcohol, caffeine, and antioxidant vitamins for 12 h before the assessment. Measurements were conducted in a temperature-controlled room with the participants in the supine position for 20 min before scanning. To ensure consistency and minimize operator-dependent bias, these measurements were performed by three experienced physicians who were strictly trained to adhere to the International Brachial Artery Reactivity Task Force guidelines. Furthermore, to standardize the hemodynamic stimulus across all assessments, baseline brachial artery diameters were measured using the built-in automated edge-detection software of the UNEXEF18G system, and FMD values were corrected for shear rate.

A high-resolution ultrasound system (UNEXEF18G; UNEX Co., Nagoya, Japan) with automated edge-detection software was used to assess the FMD of the brachial artery. Blood pressure cuffs were inflated to 50 mmHg above systolic pressure for 5 min, followed by deflation. Brachial artery diameters were recorded continuously, and FMD is expressed as a percentage change from the baseline diameter.

### 2.8. Randomization and Blinding

Randomization was performed using REDCap, with allocation concealment ensured by stratified block randomization and a block size of 3. This study was not blinded because of the practical challenges of blinding the intervention, particularly when testing food appearance.

### 2.9. Data Collection

Data were collected at baseline and at the start and end of each intervention period, covering the following:

1. Anthropometric measures (weight, body mass index [BMI], body fat percentage).
2. Laboratory parameters (e.g., blood glucose, lipid profile, trigonelline levels). To eliminate inter-laboratory variability, all routine biochemical and hematological parameters were centrally analyzed using standardized automated clinical analyzers at the Department of Clinical Laboratory, Kagoshima University Hospital.
3. FMD measurements.
4. Adherence to intervention (percentage of consumed test food packets).

#### Quantification of Specialized Biomarkers

In addition to routine laboratory tests, specialized biomarkers were quantified at a central laboratory using the following validated analytical principles:

- Urinary nitric oxide metabolites and oxidative stress markers: Urinary levels of nitric oxide metabolites (NOx; nitrite/nitrate) were quantified using a standardized colorimetric assay based on the Griess reaction following enzymatic nitrate reduction (Nitrate/Nitrite Colorimetric Assay Kit, Cayman Chemical, Ann Arbor, MI, USA). Urinary 8-hydroxy-2′-deoxyguanosine (8-OHdG) was measured using a competitive enzyme-linked immunosorbent assay (ELISA) and normalized to urinary creatinine levels (ng/mg Cr) to correct for variations in urine concentration (New 8-OHdG Check ELISA, Japan Institute for the Control of Aging, Fukuroi, Shizuoka, Japan). Serum thiobarbituric acid reactive substances (TBARS), an index of systemic lipid peroxidation, were quantified using a standardized fluorometric/colorimetric assay (TBARS Microplate Assay Kit, Oxford Biomedical Research Inc., Rochester Hills, MI, USA).
- Inflammatory markers: Serum pentraxin 3 (PTX3) was quantified using a standard ELISA (Human PTX3/Pentraxin 3 ELISA Kit, PicoKine, Boster Biological Technology, Pleasanton, CA, USA).
- Vascular function and metabolic parameters: Serum asymmetric dimethylarginine (ADMA) was measured using a commercial ELISA kit from Immundiagnostik AG (Bensheim, Germany), and serum niacin was measured using the ID-Vit Niacin kit (Immundiagnostik AG, Bensheim, Germany).

All were measured according to the respective manufacturers’ instructions.

### 2.10. Statistical Analyses

The sample size was determined by detecting a 2% improvement in %FMD, with 90% power at a 5% significance level, considering an estimated standard deviation of 1.9. Based on these parameters for a crossover design, a minimum of 12 participants was required. To account for potential dropouts and feasibility, we planned to enroll a total of 21 participants.

Descriptive statistics were calculated for baseline characteristics; continuous variables are presented as means and standard deviations or medians and interquartile ranges, and categorical variables are presented as numbers and percentages. For the primary outcome (%FMD improvement), linear mixed-effects models, including fixed effects for time, treatment, period, and cross-product terms between time and treatment as covariates and with a random intercept for participants, were used to account for within-subject correlations. To assess potential carryover effects, a first-order carryover term (representing the treatment administered in the immediately preceding period) was included in the model. Secondary outcomes were descriptively analyzed using exploratory inferential methods. Statistical significance was set at a two-sided *p*-value < 0.05. All analyses were performed using the R software, version 4.4.0 (R Foundation for Statistical Computing; R Foundation, Vienna, Austria) [9].

### 2.11. Ethical Considerations

The study protocol was approved by the Clinical Research Review Board of Kagoshima University Hospital and conducted in accordance with the Declaration of Helsinki. The study protocol was registered in the Japan Registry of Clinical Trials (jRCT) on 30 March 2022 under the identifier jRCT s071210142.

Written informed consent was obtained from all the participants. Adverse events were monitored and reported according to the regulatory guidelines. Financial compensation for transportation and other inconveniences was also provided.

This comprehensive methodology ensures transparency, reproducibility, and alignment with CONSORT standards (Figure 1).

## 3. Results

### 3.1. Overview of the Study Participants

Twenty-four participants consented to join this study, of whom three were excluded for not meeting the eligibility criteria (one owing to ongoing treatment for a chronic disease and two without MetS). Ultimately, 21 participants were enrolled and randomized equally into three sequences (sequences 1, 2, and 3 with 7 participants each). All participants completed the intervention, and all datasets were included in the Full Analysis Set (FAS), Per Protocol Set (PPS), and Safety Analysis Set (SAS) analyses.

### 3.2. Baseline Characteristics

#### Patient Characteristics (All Participants [n = 21])

Twenty-one patients participated in this study, consisting of 12 (57.14%) females and 9 (42.86%) males. The baseline demographic and clinical characteristics, including anthropometric measurements, underlying comorbidities, and concomitant medications, are summarized in Table 1. Baseline hematological and biochemical parameters, demonstrating the foundational metabolic and inflammatory status of the participants, are detailed separately in Table 2.

The mean age was 59.33 years (standard deviation [SD], 8.82; age min–max, 42–73 years).

The mean height was 161.56 cm (SD, 9.62), and the mean weight was 71.50 kg (SD, 9.98). The mean BMI was 27.34 kg/m^2^ (SD, 2.42), indicating a tendency toward obesity, and the mean body fat percentage was 29.38% (SD, 5.30).

Hypertension was the most prevalent metabolic condition in 19 (90.5%) patients, followed by diabetes mellitus (n = 8, 38.10%) and dyslipidemia (n = 14, 66.7%). The treatment approaches included observation, dietary therapy, and exercise therapy.

Statins and angiotensin receptor blockers (ARBs) were the most commonly prescribed medications, with 12 (57.14%) patients taking these medications. Patients with hypertension were treated with angiotensin-converting enzyme (ACE) inhibitors, calcium channel blockers, and diuretics, whereas those with diabetes were treated with metformin, dipeptidyl peptidase-4 (DPP-4) inhibitors, and sodium-glucose cotransporter 2 (SGLT2) inhibitors. Only one (4.76%) patient used fibrate derivatives, and there were no cases of ezetimibe or omega-3 fatty acid use.

Blood test results showed a mean total cholesterol (TC) level of 205.43 mg/dL (SD, 34.88) and high-density lipoprotein (HDL) cholesterol of 67.52 mg/dL (SD, 17.26). Liver function tests were within the normal range, with a mean aspartate aminotransferase (AST) level of 24.40 U/L (SD, 8.22) and an alanine aminotransferase (ALT) level of 27.53 U/L (SD, 12.57). The mean serum creatinine level was 0.80 mg/dL (SD, 0.23), indicating preserved renal function.

Sequence 1: Usual → Sakurajima → Aokubi (n = 7)

Seven patients were allocated to Sequence 1, consisting of two (28.57%) females and five (71.43%) males. The mean age was 60.57 (SD, 7.23) years (age min–max, 47–67 years). The mean height was 165.01 cm (SD, 8.80), and the mean weight was 72.93 kg (SD, 9.96). The mean BMI was 26.69 kg/m^2^ (SD, 1.93), and the mean body fat percentage was 26.89% (SD, 4.88%). Hypertension was the most prevalent metabolic condition (n = 6, 85.71%), followed by diabetes mellitus (n = 3, 42.86%) and dyslipidemia (n = 7, 100.00%). Regarding treatment approaches, although there were no patients under observation only, one (14.29%) patient received dietary and exercise therapy, and six (85.71%) patients received pharmacological therapy. Regarding medication status, statins were prescribed to five (71.43%) patients and ARBs to four (57.14%) patients. For hypertension treatment, calcium channel blockers were prescribed to four (57.14%) patients, with no use of ACE inhibitors or diuretics. For diabetes treatment, two (28.57%) patients used metformin, one (14.29%) patient used DPP-4 inhibitors, and three (42.86%) patients used SGLT2 inhibitors. One (14.29%) patient used fibrate derivatives without ezetimibe or omega-3 fatty acids. Blood test results showed a mean TC of 193.29 mg/dL (SD, 33.01) and HDL cholesterol of 69.71 mg/dL (SD, 24.94). Liver function tests were within the normal range, with a mean AST level of 26.80 U/L (SD, 12.30) and an ALT level of 26.20 U/L (SD, 14.24). The mean serum creatinine level was 0.89 mg/dL (SD, 0.22), indicating preserved renal function.

Sequence 2: Sakurajima → Aokubi → Usual (n = 7)

Seven patients were allocated to Sequence 2, consisting of four (57.14%) females and three (42.86%) males. The mean age was 60.43 (SD, 10.44) years (age min–max, 48–73 years). The mean height was 160.50 cm (SD, 13.71), and the mean weight was 69.73 kg (SD, 13.99). The mean BMI was 26.87 kg/m^2^ (SD, 1.75), and the mean body fat percentage was 28.06% (SD, 3.33%). Hypertension was the most prevalent metabolic condition (n = 7, 100.00%), followed by diabetes mellitus (n = 3, 42.86%) and dyslipidemia (n = 4, 57.14%). Regarding treatment approaches, although there were no patients under observation only, one (14.29%) patient received dietary and exercise therapy, and six (85.71%) patients received pharmacological therapy. Regarding medication status, statins were prescribed to four (57.14%) patients and ARBs to three (42.86%) patients. For hypertension treatment, calcium channel blockers were prescribed to four (57.14%) patients, with no use of ACE inhibitors or diuretics. For diabetes treatment, two (28.57%) patients used metformin and two (28.57%) patients used DPP-4 inhibitors without the use of SGLT2 inhibitors. No cases of fibrate derivative use were reported. Blood test results showed a mean TC level of 201.43 mg/dL (SD, 43.15) and an HDL cholesterol level of 70.00 mg/dL (SD, 14.73). Liver function tests were within the normal range, with a mean AST level of 20.80 U/L (SD, 5.63) and an ALT level of 22.60 U/L (SD, 12.24). The mean serum creatinine level was 0.85 mg/dL (SD, 0.27), indicating preserved renal function.

Sequence 3: Aokubi → Usual → Sakurajima (n = 7)

Seven patients were allocated to Sequence 3, consisting of six (85.71%) females and one (14.29%) male. The mean age was 57.00 (SD, 9.42) years (age min–max, 42–70 years). The mean height was 159.17 cm (SD, 4.62), and the mean weight was 71.84 kg (SD, 5.58). The mean BMI was 28.47 kg/m^2^ (SD, 3.24), and the mean body fat percentage was 33.20% (SD, 5.68%). Hypertension was the most prevalent metabolic condition (n = 6, 85.71%), followed by diabetes mellitus (n = 2, 28.57%) and dyslipidemia (n = 3, 42.86%). Regarding treatment approaches, one (14.29%) patient was under observation only, with no implementation of dietary or exercise therapy. Regarding medication status, statins were prescribed to three (42.86%) patients and ARBs to five (71.43%) patients. For hypertension treatment, calcium channel blockers were prescribed to three (42.86%) patients and diuretics to one (14.29%) patient, with no use of ACE inhibitors. For diabetes treatment, one (14.29%) patient used metformin, DPP-4 inhibitors, and SGLT2 inhibitors. No cases of fibrate derivative use were reported. Blood test results showed a mean TC level of 221.57 mg/dL (SD, 24.68) and an HDL cholesterol level of 62.86 mg/dL (SD, 10.79). Liver function test results were within the normal range, with a mean AST level of 25.60 U/L (SD, 5.32) and an ALT level of 33.80 U/L (SD, 10.89). The mean serum creatinine level was 0.68 mg/dL (SD, 0.16), indicating preserved renal function.

Although minor random variations in certain baseline characteristics (e.g., sex distribution) were observed across the three sequence groups due to the small sample size per sequence (n = 7), the overall demographic, clinical, and biochemical profiles were generally comparable. Crucially, because this study utilized a rigorous crossover design, each participant served as their own internal control. Consequently, any incidental between-sequence baseline imbalances are mathematically neutralized within the linear mixed-effects model, ensuring they do not confound the estimation of the primary efficacy outcomes.

### 3.3. Primary Outcome Results

#### 3.3.1. Changes in the Mean %FMD over Time

**Figure 3:**
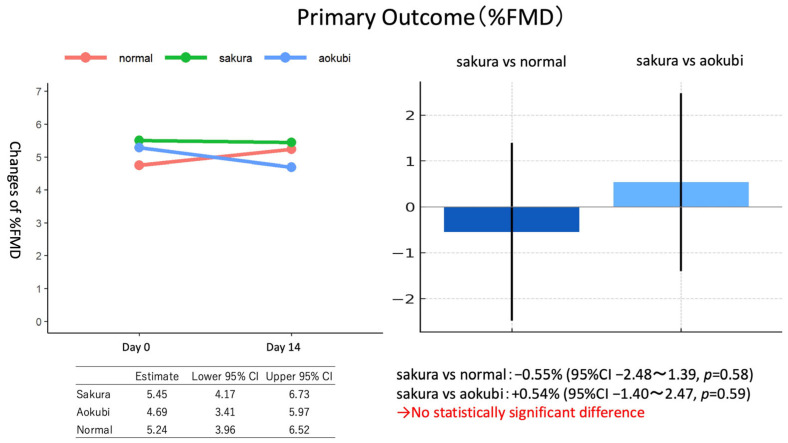
Effects of Sakurajima radish consumption on the primary outcome (flow-mediated dilation; %FMD) in patients with metabolic syndrome. FMD, flow-mediated dilation; CI, confidence interval.

The analysis of the mean %FMD values across different dietary interventions revealed that the Sakurajima radish powder group had a mean %FMD of 5.45 (Figure 3).

The left panel presents the longitudinal changes and model-estimated marginal means of %FMD across the three 14-day intervention periods (Sakurajima radish, Aokubi radish, and Usual diet). The accompanying table displays the estimated mean values with their corresponding 95% confidence intervals (CIs). The right panel illustrates the comparative treatment effects, expressed as between-group mean differences in %FMD. Error bars denote the 95% CIs. Statistical analyses using a linear mixed-effects model—accounting for within-subject correlations, period effects, and treatment effects inherent to the crossover design—revealed no significant improvement in %FMD following the Sakurajima radish intervention compared to the Usual diet (−0.55%; 95% CI, −2.48 to 1.39; *p* = 0.58) or the Aokubi radish active control (0.54%; 95% CI, −1.40 to 2.47; *p* = 0.59).

The 95% confidence interval (CI) for this value ranged from 4.17 to 6.73, indicating variability within this range. In the Aokubi radish powder group, the mean %FMD was 4.69, with a 95% CI ranging from 3.41 to 5.97. This suggests that, on average, participants in this group exhibited slightly lower %FMD values than those in the Sakurajima radish powder group. In the usual diet group, the mean %FMD was 5.24, with a 95% CI ranging from 3.96 to 6.52. This value was intermediate between the two radish groups, with some overlap in the CIs for both intervention groups. These findings indicate that the mean %FMD values are relatively similar across the three groups, with the Sakurajima radish powder group showing a slightly higher mean %FMD than the Aokubi radish powder and usual diet groups.

#### 3.3.2. Changes in FMD

The results showed no significant improvement in vascular endothelial function, as indicated by %FMD. Specifically, when comparing the Sakurajima radish group to the usual diet group, the effect size was −0.55, with a 95% CI ranging from −2.48 to 1.39. The *p*-value was 0.58, indicating no statistically significant difference between the groups.

Similarly, when comparing the Sakurajima radish group to the Aokubi radish group, the effect size was 0.54, with a 95% CI ranging from −1.40 to 2.47. The *p*-value for this comparison was 0.59, indicating no statistically significant difference.

### 3.4. Exploratory Analysis of the Effects of Phases 1, 2, and 3 on %FMD Changes

To assess whether the timing of the dietary intervention influences %FMD changes, an exploratory analysis was performed by comparing the effects observed in phases 2 and 3 against phase 1 as the reference period (Table 3).

The results showed that in phase 2, the %FMD was higher by 0.48 units compared with phase 1. However, the CI for this effect ranged from −0.88 to 1.85, suggesting considerable variability in the data. The *p*-value for this comparison was 0.51, indicating that the increase is not statistically significant.

Conversely, in phase 3, the %FMD was lower by 0.74 units compared with that in phase 1. The 95% CI for this effect ranged from −2.06 to 0.60, demonstrating a wide range of potential values. The *p*-value was 0.30, suggesting that the decrease is not statistically significant.

Overall, these findings indicate that the timing of the intervention does not have a statistically significant effect on %FMD changes. The variability observed in the CIs suggests that individual differences or external factors may have influenced the results.

### 3.5. Exploratory Analysis of the Carryover Effects of Sakurajima and Aokubi Radishes on %FMD

An exploratory analysis was performed to assess the potential carryover effects of prior radish intake on %FMD (Table 4).

The results showed that the consumption of Sakurajima radish in the previous period resulted in a change in %FMD of −1.0. The 95% CI for this effect ranged from −2.51 to 0.51, and the *p*-value was 0.20, indicating that this effect is not statistically significant.

However, the consumption of Aokubi radish in the previous period led to a change in %FMD of −2.0. The 95% CI ranged from −3.51 to −0.49, and the *p*-value was 0.01, indicating a statistically significant negative carryover effect.

These findings suggest that prior consumption of Aokubi radish may have a lasting impact on endothelial function, potentially reducing %FMD measurements in subsequent periods. In contrast, the carryover effect of Sakurajima radish was not statistically significant, suggesting that its influence on %FMD does not persist beyond the immediate consumption period.

### 3.6. Secondary Outcome Results

Serum trigonelline levels were measured as a predefined secondary outcome using HPLC. However, because a high proportion of samples were unquantifiable or missing under the assay conditions used in this trial, robust statistical comparison was not feasible. The available exploratory data, representing quantifiable samples only, are presented in Supplementary Table S1.

#### 3.6.1. Blood Pressure

Blood pressure changes were analyzed across the dietary interventions (Table 5).

The Sakurajima radish group showed significantly higher systolic blood pressure (SBP) compared with both the usual diet (mean difference, 9.67 mmHg; 95% CI, 1.16–18.18; *p* = 0.03) and Aokubi radish (8.86 mmHg; 95% CI, 0.35–17.37; *p* = 0.04) groups. DBP changes were not statistically significant, with increases of 5.57 mmHg vs. the usual diet (*p* = 0.06) and 4.62 mmHg vs. Aokubi radish (*p* = 0.12).

These findings indicate that Sakurajima radish significantly increases SBP compared with other interventions, whereas its effect on DBP was inconclusive.

#### 3.6.2. Lipid Profile

Changes in lipid profiles were also analyzed. The Sakurajima radish group had lower TC levels compared with the usual diet group, with a mean difference of −5.57 mg/dL (95% CI, −17.23–6.09; *p* = 0.35), and the Aokubi radish group, with a mean difference of −7.57 mg/dL (95% CI, −19.23–4.09; *p* = 0.21). However, these differences were not statistically significant. The Sakurajima radish group had a nonsignificantly lower low-density lipoprotein cholesterol level (–2.14 mg/dL) (95% CI, −13.14–8.86; *p* = 0.70) compared with the usual diet and Aokubi radish (–3.29 mg/dL [95% CI, −14.29–7.71; *p* = 0.56]) groups. The Sakurajima radish group had slightly lower HDL cholesterol levels compared with both the usual diet (–1.71 mg/dL; 95% CI, −5.58–2.15; *p* = 0.39) and Aokubi radish (–2.05 mg/dL; 95% CI, −5.91–1.82; *p* = 0.30) groups, although these changes were not statistically significant. The Sakurajima radish group had lower triglyceride levels (–18.14 mg/dL; 95% CI, −46.98–10.70; *p* = 0.22) compared with the usual diet and Aokubi radish (–14.81 mg/dL; 95% CI, −43.65–14.03; *p* = 0.32) groups, but neither reached statistical significance.

#### 3.6.3. Inflammatory Markers

The Sakurajima radish group had a nonsignificant lower high-sensitivity troponin I level (–0.10 ng/L; 95% CI, −0.52–0.32; *p* = 0.64) compared with the usual diet and Aokubi radish groups (–0.36 ng/L; 95% CI, −0.78–0.06; *p* = 0.10). For pentraxin 3, the Sakurajima radish group showed a highly variable effect with 1068.81 pg/mL (95% CI, −176,693.11–178,830.73; *p* = 0.99) compared with the usual diet and Aokubi radish (111,998.48 pg/mL; 95% CI, −65,763.44–289,760.40; *p* = 0.22) groups, indicating no significant difference.

#### 3.6.4. Urinary Nitric Oxide Metabolites and Oxidative Stress Markers

Changes in the oxidative stress markers were analyzed. The Sakurajima radish group had a significantly higher urinary NOx level compared to the usual diet group (769.05 µmol/L; 95% CI, 94.36–1443.75; *p* = 0.03), whereas the comparison with the Aokubi radish group showed no significant difference (–490.66 µmol/L; 95% CI, −1165.36–184.03; *p* = 0.16). The Sakurajima radish group had a significantly higher urinary 8-hydroxy-2′-deoxyguanosine (8-OHdG)/creatinine ratio compared with the usual diet (2.74 ng/mg Cr; 95% CI, 0.54–4.95; *p* = 0.02), whereas the difference with the Aokubi radish group was not significant (0.67 ng/mg Cr; 95% CI, −1.53–2.88; *p* = 0.55). Furthermore, serum TBARS, another marker of systemic oxidative stress (lipid peroxidation), showed no significant difference between the Sakurajima radish group and the usual diet (10.25 nmol/mL; 95% CI, −10.49–30.99; *p* = 0.34) or Aokubi radish (7.55 nmol/mL; 95% CI, −13.19–28.29; *p* = 0.48) groups.

#### 3.6.5. Vascular Function Markers

Changes in vascular function markers were also analyzed. For asymmetric dimethylarginine, the Sakurajima radish group showed no significant difference compared with the usual diet (–0.01 µmol/L; 95% CI, −0.05–0.03; *p* = 0.73) and Aokubi radish n (−0.01 µmol/L; 95% CI, −0.05–0.03; *p* = 0.69) groups.

#### 3.6.6. Nutritional and Metabolic Parameters

Regarding systemic nutritional parameters, serum niacin levels in the Sakurajima radish group showed no significant difference compared with the usual diet (0.76 µmol/L; 95% CI, −6.36–7.88; *p* = 0.83) or Aokubi radish (–2.84 µmol/L; 95% CI, −9.96–4.28; *p* = 0.44) groups.

### 3.7. Sensory Evaluation Results

The Sakurajima radish group had higher mean scores for sweetness (3.81 vs. 2.90) and umami (3.43 vs. 3.00) than the usual diet and Aokubi radish groups (Table 6).

Conversely, the Aokubi radish group had higher mean scores for bitterness, pungency, and astringency than the Sakurajima radish group. Palatability scores were comparable between the two groups.

### 3.8. Adverse Events

The adverse events were generally mild, and no severe adverse events were reported in any group (Table 7).

The most common events were transient gastrointestinal symptoms (e.g., abdominal bloating and mild diarrhea), which resolved spontaneously without intervention discontinuation.

## 4. Discussion

This phase IIb, open-label, randomized, three-sequence, three-period crossover trial aimed to evaluate the efficacy and safety of Sakurajima radish powder, known for its high trigonelline content, on vascular endothelial function in patients with MetS. Contrary to our hypothesis based on promising phase I data in healthy individuals [7], the primary outcome analysis revealed that Sakurajima radish consumption for 14 days did not significantly improve FMD (%FMD) compared with either a control diet period or the consumption of Aokubi radish powder. Furthermore, several unexpected secondary findings emerged, including a significant increase in SBP and a marker of oxidative DNA damage (urinary 8-OHdG), with Sakurajima radish intervention, along with a significant increase in urinary NO metabolites (NOx).

### 4.1. Lack of Effect on the Primary Outcome (FMD)

The lack of improvement in %FMD, the primary measure of endothelial function, contrasts with a previous single-arm study that reported FMD enhancement with Sakurajima radish in healthy volunteers [7]. Several factors may have contributed to the discrepancy. First, the study populations differ significantly. Compared with healthy individuals, patients with MetS often exhibit established endothelial dysfunction, which is potentially resistant to short-term dietary interventions [3,4]. The coexisting conditions (hypertension, diabetes, dyslipidemia) and concomitant medications (statins, ARBs, antihypertensives, antidiabetics) prevalent in our cohort can also influence endothelial responsiveness or interact with the intervention [3]. Although the crossover design controls for stable inter-individual differences, these underlying factors may blunt the potential benefits observed in a healthier population. Second, the intervention duration of 14 days, although sufficient based on assumed pharmacokinetics for washout, might be significantly short to induce significant structural or functional improvements in endothelial health in patients with MetS [10]. Longer intervention periods may be necessary to observe changes in FMD. Third, despite standardized protocols, inherent variability in FMD measurements can affect the statistical power, particularly for smaller sample sizes [11,12]. Although our sample size calculation targeted a 2% FMD improvement with 90% power, the observed effect size was negligible (–0.55% vs. control), suggesting either a true lack of effect or variability masking a smaller potential effect.

### 4.2. Interpretation of Secondary and Unexpected Findings (Urinary Nitric Oxide Metabolites, Systolic Blood Pressure, 8-Hydroxy-2′-deoxyguanosine)

Notably, despite the neutral effect on FMD, Sakurajima radish consumption led to significantly higher urinary NOx levels compared with the control diet. Urinary NOx levels reflect systemic NO production and metabolism [13]. This finding suggests that Sakurajima radish enhances NO synthesis and bioavailability, potentially through its high trigonelline and other bioactive compound contents. The significant increase in urinary NOx levels confirms that Sakurajima radish successfully stimulated NO synthesis in MetS patients, likely via the aforementioned eNOS activation pathway (phosphorylation at Ser1177).

However, this increased NO production did not translate into improved brachial artery vasodilation, as measured using FMD. This dissociation could imply that increased NO was rapidly scavenged (possibly indicated by the concurrent increase in oxidative stress) and utilized in other pathways or that the vascular smooth muscle response to NO was impaired in patients with MetS [14]. Regarding the source of increased NOx, while we cannot definitively rule out the contribution of dietary nitrates/nitrites from the Sakurajima radish, the observed clinical outcomes—specifically the lack of FMD improvement and the rise in oxidative stress markers—strongly suggest that this NOx elevation is not synonymous with increased bioavailable NO for vascular relaxation. Rather, we hypothesize a scenario where robust NO synthesis occurs, but the subsequent biochemical partitioning is dominated by the formation of peroxynitrite in the MetS milieu, a hypothesis supported by the concurrent elevation in 8-OHdG and SBP. The significant increase in the levels of urinary 8-OHdG/creatinine, a marker of oxidative DNA damage [15], with Sakurajima radish consumption compared with the control is concerning and counterintuitive for an intervention hypothesized to improve vascular health. Although some plant phytochemicals can exhibit prooxidant effects at certain doses or under specific metabolic conditions [16], the mechanism is unclear. This paradoxical finding can be pathophysiologically explained by the concomitant elevation of oxidative stress, evidenced by the significant increase in urinary 8-OHdG levels [15]. In the highly oxidative environment typical of MetS, the robustly produced NO is prone to react with superoxide (O_2_^−^), forming peroxynitrite (ONOO^−^). This reaction not only depletes bioavailable NO (preventing vasodilation) but also promotes further oxidative DNA damage and vascular smooth muscle constriction, thereby potentially driving the unexpected elevation in systolic blood pressure observed in our study. This warrants caution and further investigation, especially regarding the potential interplay between trigonelline and other radish components and the proinflammatory/oxidative state characteristics of MetS.

Another unexpected finding was the significant elevation in SBP in the Sakurajima radish group compared with that in both the control and Aokubi radish groups. Consistent with this potential vasoconstrictive effect of peroxynitrite, a significant elevation in SBP was observed in the Sakurajima radish group compared with that in both the control and Aokubi radish groups. This contrasts with some studies suggesting the potential blood pressure-lowering effects of dietary nitrates (abundant in many vegetables) or trigonelline in other contexts [17,18]. However, the effects of trigonelline on blood pressure are not fully established, and other compounds in Sakurajima radish can potentially exert hypertensive effects or interact with the participants’ existing medications [19]. This finding underscores the importance of comprehensive safety assessments in functional food trials.

### 4.3. Methodological Considerations (Carryover Effect)

Aokubi radish was used as an active control because of its lower trigonelline content than Sakurajima radish. Although Sakurajima did not significantly improve %FMD compared with Aokubi, exploratory analysis detected a significant negative carryover effect for Aokubi (*p* = 0.01). This indicates that Aokubi consumption reduces %FMD in the subsequent period, suggesting that the 14-day washout period may have been insufficient to eliminate its biological effects.

Importantly, the sequence structure showed that Aokubi was followed by the usual diet period in two of the three sequences, and no sequence included Aokubi immediately followed by Sakurajima. Therefore, the negative carryover from Aokubi likely affected the subsequent control period, rather than the Sakurajima period.

Consequently, the %FMD values during some control periods may have been artificially lowered, indicating that the control condition may not have consistently represented a true baseline state. Although the mixed-effects model adjusts for period effects, this structural imbalance could have influenced the treatment comparisons involving the control group.

No significant carryover was observed for Sakurajima radish.

### 4.4. Critical Appraisal: Unpacking the Paradoxical Hemodynamic Response

Our divergent findings—specifically, elevated urinary NOx levels without concurrent improvement in flow-mediated dilation (FMD), alongside unexpected elevations in systolic blood pressure (SBP) and oxidative stress markers (8-OHdG)—warrant a careful mechanistic appraisal. We hypothesize that for dietary phytochemicals, the dose–response relationship follows a non-linear trajectory; while moderate exposure may facilitate NO-mediated vasodilation, our intervention dose of 10 g/day, within the highly pro-oxidant milieu characteristic of metabolic syndrome (MetS), may have surpassed the therapeutic threshold, triggering a maladaptive response. In this state, robustly produced NO likely reacts with ambient superoxide (O_2_^−^) to form peroxynitrite (ONOO^−^), a potent oxidant that promotes eNOS uncoupling, thereby exacerbating oxidative damage rather than inducing vasodilation. Furthermore, the crossover design may have inadvertently magnified these adverse hemodynamic effects. Given the significant carryover phenomenon observed, the repeated, structured exposure to high-dose trigonelline over three periods likely acted as a cumulative biological stressor, sensitizing patients to these hemodynamic shifts through sequence-dependent persistent effects. This carryover phenomenon fundamentally limited our ability to isolate the specific therapeutic impact of the Sakurajima radish, as persistent physiological alterations likely confounded the baseline status of subsequent periods, thereby potentially obscuring the true therapeutic window of our intervention. Consequently, these results underscore that high-dose NO-boosting interventions in patients with MetS require extreme caution. Our data suggest that future parallel-group randomized controlled trials are essential to rigorously define the safe and therapeutic dosage of Sakurajima radish, ensuring that clinical interventions prioritize the avoidance of eNOS uncoupling and oxidative injury over the simple augmentation of systemic NO synthesis.

### 4.5. Strengths and Limitations

The strengths of this study include its randomized controlled design, the use of a crossover methodology to reduce inter-individual variability, the inclusion of both placebo (control diet) and active (Aokubi radish) comparators, and standardized FMD measurements. Furthermore, translating dietary interventions into real-world practice requires high patient adherence. Our sensory evaluation (Table 5) confirmed that Sakurajima radish is highly palatable, comparable to the standard Aokubi radish. This demonstrates its tolerability and real-world feasibility for long-term daily consumption without compromising compliance.

However, some limitations warrant consideration when interpreting the generalizability of our findings. First, the intervention period of 14 days, while pharmacokinetically sufficient for washout based on the trigonelline half-life, may have been significantly short to induce structural vascular remodeling or significant functional improvement in patients with MetS. Unlike healthy volunteers, endothelial dysfunction in patients with MetS is often chronic and may exhibit resistance to short-term dietary interventions owing to “metabolic memory,” necessitating longer durations to reverse established vascular impairment. Second, the background optimal medical therapy (e.g., antihypertensive, lipid-lowering, and antidiabetic agents) prescribed to the patients may have optimized baseline endothelial function, creating a ceiling effect that masked the specific physiological benefits of the dietary intervention. While excluding medicated patients from the protocol would have minimized this confounding, we deliberately included them to reflect real-world clinical practice. This pragmatic design choice was essential to preserve the external validity and generalizability of our findings for typical patients with metabolic syndrome, as well as to maintain strict ethical standards regarding the continuation of standard-of-care treatments. However, we acknowledge that this design choice precludes a clear statistical adjustment for the individual effects of these medications, which may contribute to the heterogeneity of the FMD response and potentially limit our ability to isolate the independent effect of the intervention. Third, although the sample size was calculated based on prior data, the enrollment of 21 participants may have been underpowered to account for the heterogeneous nature of FMD responses in a pathologically diverse MetS cohort; thus, the possibility of a type II error cannot be ruled out. Fourth, the open-label design necessitated by the distinct visual characteristics of the test foods introduces potential bias, although the use of automated edge-detection software for FMD mitigates subjective measurement errors. Furthermore, a significant negative carryover effect on FMD was observed following the Aokubi radish period. We must explicitly acknowledge that the presence of such carryover effects fundamentally limits causal inference within this crossover design, as it may attenuate or distort the true treatment contrasts between the sequences. Consequently, the true physiological efficacy of the Sakurajima radish may have been masked by these methodological constraints, emphasizing the necessity for future parallel-group randomized trials to eliminate such confounding. From a clinical perspective, these observations indicate that the washout period was insufficient to fully reset the physiological status, thereby confounding subsequent baseline measurements. Therefore, the lack of significant FMD improvement in our crossover model should not be interpreted as definitive clinical evidence of inefficacy. Instead, this result highlights the high sensitivity of endothelial function to preceding metabolic interventions in patients with MetS and underscores the potential for carryover-induced bias to obscure subtle therapeutic effects. Consequently, our findings should be interpreted as an exploratory insight into the complex vascular dynamics in MetS, strongly advocating for future parallel-group randomized controlled trials to definitively determine the clinical utility of Sakurajima radish. Finally, the underlying mechanisms driving divergent outcomes, specifically the increase in urinary NOx levels without concurrent FMD improvement, accompanied by unexpected elevations in SBP and oxidative stress marker levels (8-OHdG), remain unclear. The potential for NO synthase uncoupling or other biochemical interactions in the context of MetS requires further investigation to fully understand the safety profile and physiological impact of high-trigonelline radish consumption. Fifth, we could not establish a direct pharmacokinetic–pharmacodynamic (PK-PD) relationship. Serum trigonelline concentrations were measured using a dedicated HPLC bioanalytical assay for serum samples, as detailed in Supplementary Table S1. This method was distinct from the HPLC-based plant-tissue compositional analyses used in our prior publications to quantify trigonelline content in Sakurajima and Aokubi radishes. However, a high proportion of serum samples were below the quantification limit or missing under the assay conditions used in the present trial, precluding reliable pharmacokinetic analysis. To maximize real-world adherence, participants were permitted to consume the radish powder either dissolved in water or incorporated into meals; therefore, the administration conditions were not strictly standardized. Differences in the administration matrix, timing of intake, meal composition, gastric emptying, and intestinal absorption conditions may have introduced substantial intra- and inter-individual variability in absorption kinetics and systemic exposure to trigonelline. This food-matrix-related variability, together with the analytical sensitivity limitations and sampling conditions of the serum assay, may have contributed to the fluctuating or unquantifiable serum trigonelline concentrations and further complicated the evaluation of its physiological efficacy. In addition, our dosage rationale relied on literature-based trigonelline content estimates rather than batch-specific analytical verification of the exact investigational powder lots used in this trial. Therefore, the estimated trigonelline intake should be interpreted as a nominal dose rather than a directly measured batch-specific or confirmed bioavailable dose. Future studies should incorporate concurrent batch-specific compositional testing of the investigational food product, strictly standardized administration protocols under fasting or controlled meal conditions, and a validated, more sensitive bioanalytical method, such as LC-MS/MS, to accurately quantify circulating trigonelline and assess its relationship with vascular outcomes. Sixth, we did not collect detailed dietary records (e.g., food frequency questionnaires or 24 h recalls) during the study. Although the crossover design and instructions to maintain baseline dietary habits were intended to minimize intra-individual variability, unmeasured fluctuations in the intake of other vasoactive nutrients (such as background dietary nitrates or sodium) cannot be completely ruled out as potential confounders in this free-living trial. Seventh, regarding the investigational product, we did not perform batch-specific quantitative analysis of the trigonelline content for the exact powder lots used in this trial. Although our intervention was justified based on our previously established analytical evidence confirming the trigonelline-rich nature of the Sakurajima radish [7], the lack of real-time quantification of the test foods limits our ability to calculate the exact daily dose of trigonelline administered to each patient. Finally, FMD measurements were performed by three independent physicians. Although all operators underwent rigorous training to standardize the acquisition protocol according to international guidelines, we cannot entirely rule out the potential for inter-observer variability. While we utilized automated edge-detection software and shear rate correction to mitigate operator-dependent measurement bias, the lack of a formal pre-study inter-observer reproducibility assessment remains a methodological limitation. Future multicenter studies should incorporate centralized core-lab analysis to further minimize such operator-related variance.

## 5. Conclusions

In conclusion, this exploratory phase IIb crossover trial did not demonstrate a beneficial effect of a 14-day Sakurajima radish intervention on vascular endothelial function (FMD) in patients with MetS. The observed elevations in surrogate markers—specifically urinary NOx, 8-OHdG, and systolic blood pressure—suggest that while the intervention likely stimulated NO synthesis, it elicited a complex hemodynamic response rather than straightforward therapeutic vasodilation. We hypothesize that under the highly pro-oxidant milieu of MetS, these short-term biochemical fluctuations may reflect mild eNOS uncoupling. Importantly, as this study evaluated only surrogate endpoints over a brief 14-day period, these observations cannot be extrapolated to imply long-term cardiovascular harm or disease promotion. Rather, these findings underscore the necessity for caution and highlight the need for future parallel-group, dose-finding trials of extended duration to carefully evaluate the optimal therapeutic window and safety profile of NO-boosting functional foods in high-risk populations.

## Figures and Tables

**Table 1:** Baseline demographic and clinical characteristics.

Variables	Overall	Sequence 1	Sequence 2	Sequence 3
Age	59.3 ± 8.8	60.6 ± 7.2	60.4 ± 10.4	57.0 ± 9.4
Male	9 (42.9%)	5 (71.4%)	3 (42.9%)	1 (14.3%)
Female	12 (57.1%)	2 (28.6%)	4 (57.1%)	6 (85.7%)
Height (cm)	161.6 ± 9.6	165.0 ± 8.8	160.5 ± 13.7	159.2 ± 4.6
Weight (kg)	71.5 ± 10.0	72.9 ± 10.0	69.7 ± 14.0	71.8 ± 5.6
BMI (kg/m^2^)	27.3 ± 2.4	26.7 ± 1.9	26.9 ± 1.8	28.5 ± 3.2
Body fat percentage (%)	29.4 ± 5.3	26.9 ± 4.9	28.1 ± 3.3	33.2 ± 5.7
Body temperature (°C)	36.4 ± 0.3	36.4 ± 0.2	36.4 ± 0.4	36.6 ± 0.4
Comorbidities (%)				
Diabetes	8 (38.1)	3 (42.9)	3 (42.9)	2 (28.6)
Observation	1 (4.8)	0 (0.0)	0 (0.0)	1 (14.3)
Dietary and exercise therapy	1 (4.8)	1 (14.3)	0 (0.0)	0 (0.0)
Drug therapy	7 (33.3)	3 (42.9)	3 (42.9)	1 (14.3)
Hypertension	19 (90.5)	6 (85.7)	7 (100)	6 (85.7)
Observation	0 (0.0)	0 (0.0)	0 (0.0)	0 (0.0)
Dietary and exercise therapy	1 (4.8)	0 (0.0)	1 (14.3)	0 (0.0)
Drug therapy	18 (85.7)	6 (85.7)	6 (85.7)	6 (85.7)
Dyslipidemia	14 (66.7)	7 (100.0)	4 (57.1)	3 (42.9)
Observation	0 (0.0)	0 (0.0)	0 (0.0)	0 (0.0)
Dietary and exercise therapy	1 (4.8)	1 (14.3)	0 (0.0)	0 (0.0)
Drug therapy	13 (61.9)	6 (85.7)	4 (57.1)	3 (42.9)
Medicine (%)				
Foods for specified health uses (FOSHU)	0 (0.0)	0 (0.0)	0 (0.0)	0 (0.0)
Foods with function claims	2 (9.5)	1 (14.3)	1 (14.3)	0 (0.0)
Supplement	4 (19.0)	2 (28.6)	2 (28.6)	0 (0.0)
Ezetimibe	0 (0.0)	0 (0.0)	0 (0.0)	0 (0.0)
Statin	12 (57.1)	5 (71.4)	4 (57.1)	3 (42.9)
Omega-3 fatty acid preparations	0 (0.0)	0 (0.0)	0 (0.0)	0 (0.0)
Fibrate drugs	1 (4.8)	1 (14.3)	0 (0.0)	0 (0.0)
Angiotensin II receptor blocker	12 (57.1)	4 (57.1)	3 (42.9)	5 (71.4)
Angiotensin-converting enzyme inhibitor	0 (0)	0 (0.0)	0 (0)	0 (0)
Calcium channel blocker	11 (52.4)	4 (57.1)	4 (57.1)	3 (42.9)
Nitrate drugs	0 (0.0)	0 (0.0)	0 (0.0)	0 (0.0)
Diuretics	1 (4.8)	0 (0.0)	0 (0.0)	1 (14.3)
Metformin	5 (23.8)	2 (28.6)	2 (28.6)	1 (14.3)
DPP-4 inhibitor	4 (19.0)	1 (14.3)	2 (28.6)	1 (14.3)
SGLT2 inhibitor	4 (19.0)	3 (42.9)	0 (0.0)	1 (14.3)

BMI, body mass index; DPP-4, dipeptidyl peptidase-4; FOSHU, foods for specified health uses; SGLT2, sodium-glucose cotransporter 2. Normally distributed continuous variables are presented as means ± standard deviations. Non-normally distributed variables (e.g., triglycerides, γ-GTP, high-sensitivity CRP) are presented as medians (interquartile ranges [Q1–Q3]). Categorical variables are presented as numbers (%).

**Table 2:** Baseline laboratory and biochemical parameters.

Variables	Overall	Sequence 1	Sequence 2	Sequence 3
Laboratory data				
WBC count (10^3^/μL)	6.3 ± 1.7	5.3 ± 1.0	6.6 ± 1.9	7.0 ± 1.8
RBC count (10^3^/μL)	4.7 ± 0.6	4.7 ± 0.6	4.9 ± 0.6	4.6 ± 0.4
Hemoglobin (g/dL)	14.2 ± 1.7	14.4 ± 1.7	14.9 ± 1.7	13.4 ± 1.7
Hematocrit (%)	43.3 ± 4.8	43.4 ± 5.0	44.9 ± 4.7	41.4 ± 4.7
Platelet count (10^3^/μL)	239.0 ± 59.4	214.0 ± 75.0	241.4 ± 48.1	261.4 ± 49.9
γ-GTP (U/L)	55.0 (21.0–67.0)	39.4 (17.0–64.0)	55.0 (26.0–57.5)	62.0 (36.0–180.5)
ALP (U/L)	80.9 ± 29.4	67.3 ± 26.8	88.0 ± 26.3	87.3 ± 33.8
LDH (U/L)	174.7 ± 33.8	169.4 ± 47.9	173.1 ± 25.7	181.6 ± 27.6
Total bilirubin (mg/dL)	0.9 ± 0.4	1.0 ± 0.5	1.0 ± 0.3	0.7 ± 0.2
Direct bilirubin (mg/dL)	0.1 ± 0.0	0.1 ± 0.0	0.1 ± 0.1	0.1 ± 0.1
Indirect bilirubin (mg/dL)	0.8 ± 0.3	0.9 ± 0.4	0.9 ± 0.3	0.6 ± 0.1
Cholinesterase (U/L)	358.8 ± 55.1	345.4 ± 44.8	354.9 ± 54.4	376.1 ± 67.7
Total protein (g/dL)	7.2 ± 0.4	7.1 ± 0.5	7.3 ± 0.2	7.3 ± 0.4
Albumin (g/dL)	4.3 ± 0.3	4.3 ± 0.5	4.3 ± 0.2	4.2 ± 0.1
CK (U/L)	107.5 ± 28.1	116.6 ± 26.8	109.1 ± 19.1	96.7 ± 36.3
Calcium (mg/dL)	9.5 ± 0.3	9.3 ± 0.4	9.6 ± 0.2	9.5 ± 0.2
Serum amylase	72.7 ± 25.1	82.7 ± 32.0	69.0 ± 19.9	66.3 ± 22.5
BUN (mg/dL)	14.4 ± 3.8	15.8 ± 4.4	14.9 ± 3.2	12.5 ± 3.4
Creatinine (mg/dL)	0.8 ± 0.2	0.9 ± 0.2	0.9 ± 0.3	0.7 ± 0.2
eGFR (mL/min/1.73 m^2^)	68.9 ± 16.3	66.0 ± 15.6	64.6 ± 15.0	76.0 ± 18.1
HbA1c (NGSP) (%)	6.3 ± 0.7	6.2 ± 0.4	6.5 ± 1.0	6.3 ± 0.6
Total cholesterol (mg/dL)	205.4 ± 34.9	193.3 ± 33.0	201.4 ± 43.2	221.6 ± 24.7
LDL cholesterol (direct method) (mg/dL)	120.2 ± 28.8	106.1 ± 17.9	117.9 ± 35.7	136.6 ± 24.8
HDL cholesterol (mg/dL)	67.5 ± 17.3	69.7 ± 24.9	70.0 ± 14.7	62.9 ± 10.8
Triglyceride (mg/dL)	106.0 (78.0–165.0)	119.9 (77.0–142.0)	80.0 (70.0–125.0)	145.0 (98.5–182.5)
High-sensitivity troponin I (ng/L)	10.7 ± 3.0	10.0 ± 0.0	10.0 ± 0.0	12.0 ± 5.2
High-sensitivity CRP (mg/dL)	0.12 (0.05–0.16)	0.07 (0.04–0.13)	0.09 (0.04–0.11)	0.2 ± 0.1
Blood glucose (mg/dL)	115.3 ± 19.5	115.0 ± 12.4	119.4 ± 24.1	111.4 ± 21.4
AST (U/L)	24.4 ± 8.2	26.8 ± 12.3	20.8 ± 5.6	25.6 ± 5.3
ALT (U/L)	27.5 ± 12.6	26.2 ± 14.2	22.6 ± 12.2	33.8 ± 10.9
Urine test				
Urine urobilinogen (mg/dL)	0.0 ± 0.0	0.0 ± 0.0	0.0 ± 0.0	0.0 ± 0.0
Urine pH	6.1 ± 0.8	6.1 ± 0.9	5.9 ± 0.9	6.1 ± 0.8

ALP, alkaline phosphatase; ALT, alanine aminotransferase; AST, aspartate aminotransferase; BUN, blood urea nitrogen; CK, creatine kinase; CRP, C-reactive protein; eGFR, estimated glomerular filtration rate; GTP, gamma-glutamyl transpeptidase; HbA1c, hemoglobin A1c; HDL, high-density lipoprotein; LDH, lactate dehydrogenase; LDL, low-density lipoprotein; NGSP, National Glycohemoglobin Standardization Program; RBC, red blood cell; WBC, white blood cell. Normally distributed continuous variables are presented as means ± standard deviations. Non-normally distributed variables (e.g., triglycerides, γ-GTP, high-sensitivity CRP) are presented as medians (interquartile ranges [Q1–Q3]). Categorical variables are presented as numbers (%).

**Table 3:** The effect of the timing of dietary intervention.

	Estimate	Lower 95% CI	Upper 95% CI	*p*-Value
Phase 2 (ref. phase 1)	0.48	−0.88	1.85	0.51
Phase 3 (ref. phase 1)	−0.74	−2.06	0.60	0.30

CI, confidence interval.

**Table 4:** Carryover effects of prior radish intake on %FMD.

	Estimate	Lower 95% CI	Upper 95% CI	*p*-Value
Sakurajima	−1.00	−2.51	0.51	0.20
Aokubi	−2.00	−3.51	−0.49	0.01

CI, confidence interval.

**Table 5:** Comparison of effects on physiological parameters between groups.

Parameters	Mean Difference (Sakurajima vs. Usual)	*p*-Value	Mean Difference (Sakurajima vs. Aokubi)	*p*-Value
Blood pressure				
Systolic BP (mmHg)	9.67	0.03 *	8.86	0.04 *
Diastolic BP (mmHg)	5.57	0.06	4.62	0.12
Lipid profile				
Total cholesterol (mg/dL)	−5.57	0.35	−7.57	0.21
LDL cholesterol (mg/dL)	−2.14	0.70	−3.29	0.56
HDL cholesterol (mg/dL)	−1.71	0.39	−2.05	0.30
Triglycerides (mg/dL)	−18.14	0.22	−14.81	0.32
Inflammatory markers				
hs-TnI (ng/L)	−0.10	0.64	−0.36	0.10
PTX3 (pg/mL)	1068.81	0.99	1198.48	0.22
Oxidative stress markers				
TBARS (nmol/mL)	10.25	0.34	7.55	0.48
Vascular function				
ADMA (µmol/L)	−0.01	0.73	−0.01	0.69
Nutritional and metabolic parameters				
Niacin (µmol/L)	0.76	0.83	−2.84	0.44

BP, blood pressure; LDL, low-density lipoprotein; HDL, high-density lipoprotein; hs-TnI, high-sensitivity troponin I; PTX3, pentraxin 3; ADMA, asymmetric dimethylarginine; TBARS, thiobarbituric acid reactive substances. * *p* < 0.05.

**Table 6:** Comparison of sensory evaluation scores between the Sakurajima and Aokubi radish powders.

Sensory Attribute	Sakurajima Radish (n = 21)	Aokubi Radish (n = 21)
Sweetness	3.81 ± 1.54	2.90 ± 1.48
Sourness	2.33 ± 1.65	2.38 ± 1.53
Bitterness	2.48 ± 1.60	3.29 ± 1.62
Saltiness	2.19 ± 1.33	2.19 ± 1.40
Umami	3.43 ± 1.08	3.00 ± 1.48
Pungency	1.86 ± 1.24	2.81 ± 1.54
Astringency	1.95 ± 1.24	3.05 ± 1.75
Acridity	2.48 ± 1.83	3.33 ± 1.93
Aroma	4.29 ± 1.93	4.71 ± 1.65
Palatability	3.86 ± 1.15	3.95 ± 1.50

Values are presented as means ± standard deviations. Note: Data for the “usual diet” group were excluded from the analysis as sensory evaluation was not applicable.

**Table 7:** Adverse events (AEs).

Variable	Date of Onset	Date of Resolution	Outcome	Action Taken with Study Food	Seriousness	Severity	Causality
Gastrointestinal: Abdominal pain	26 February 2023	10 March 2023	Recovered	Dose not changed	Nonserious	Mild	Related

## Data Availability

The datasets presented in this article are not readily available because they contain commercially sensitive information related to the cultivation techniques of Sakurajima Radish. Requests to access the datasets should be directed to the corresponding author.

## Supplementary Materials

The following supporting information can be downloaded at: https://www.mdpi.com/article/10.3390/nu18111801/s1, Table S1: Serum trigonelline concentrations following the 14-day dietary interventions.

## Abbreviations

The following abbreviations are used in this manuscript:

MetS	metabolic syndrome
CVD	cardiovascular disease
NO	nitric oxide
FMD	flow-mediated vasodilation
CONSORT	Consolidated Standards of Reporting Trials
SD	standard deviation
BMI	body mass index
jRCT	Japan Registry of Clinical Trials
ARBs	angiotensin receptor blockers
ACE	angiotensin-converting enzyme
DPP-4	dipeptidyl peptidase-4
SGLT2	sodium-glucose cotransporter 2
HDL	high-density lipoprotein
AST	aspartate aminotransferase
ALT	alanine aminotransferase
TC	total cholesterol
8-OHdG	8-hydroxy-2′-deoxyguanosine
SBP	systolic blood pressure
