# Supplementary material for: Efficacy and Safety of Sakurajima Radish in Patients with Metabolic Syndrome: A Phase IIb Randomized, Three-Period Crossover Trial
Source: Nutrients. 2026 Jun 3;18(11):1801. doi: 10.3390/nu18111801 (PMC13259325; doi:10.3390/nu18111801)
Supplement: Supplementary file 1 [file nutrients-18-01801-s001.zip › nutrients-4312920-supplementary.pdf]

Supplementary Table S1: Serum trigonelline concentrations following the 14-day dietary interventions.

Group (ng/mL)	Mean	SD	Median	Q1	Q3	Max	Min	N of missing
Sakurajima	136.27	8.18	138	135.05	141.25	143.6	119.7	14
Aokubi	114.05	33.11	127.6	86.18	140.68	141.5	70.2	15
Usual	116.09	49.62	133.1	131	136	141.6	3.9	14

Max, maximum; Min, minimum; N, number; Q1, first quartile; Q3, third quartile; SD, standard deviation. High-performance liquid chromatography analysis of blood trigonelline was performed using an Extrema system (JASCO Corporation, Tokyo, Japan) equipped with a YMC-Triart PFP column (4.6 × 250 mm, 3 µm particle size; YMC Co., Ltd., Kyoto, Japan). The mobile phase consisted of 10 mM phosphate buffer (pH 2.6) at a flow rate of 1.0 mL/min. The injection volume was 10 µL, the detection wavelength was 270 nm, and the column temperature was maintained at 40 °C.
